# The impact of resistance training on heart rate variability parameters in physically active young adults

**DOI:** 10.1590/1677-5449.20240152

**Published:** 2025-04-28

**Authors:** Gabriel Marcelino Barbosa, Helyel Rodrigues Gobbo, Lucas Cezar de Oliveira, Anderson Pontes Morales, Gustavo Vieira de Oliveira

**Affiliations:** 1 Universidade Federal do Rio de Janeiro – UFRJ, Rio de Janeiro, RJ, Brasil.

**Keywords:** resistance training, heart rate, cardiovascular disease, exercise, sedentary behavior, treinamento de força, frequência cardíaca, doença cardiovascular, exercício físico, comportamento sedentário

## Abstract

**Background:**

Heart rate variability (HRV) parameters are an important indicator of cardiovascular health. While it has been well established that aerobic exercise improves HRV, the effects of resistance training on HRV remain less explored.

**Objectives:**

To compare the impact of a resistance training routine on HRV parameters in physically active young adults.

**Methods:**

This observational study included 24 participants, 12 who underwent resistance training and a control group of 12 who underwent moderate-intensity exercise. HRV was assessed during a 15-minute resting period in the supine position with a Polar RS800CX heart rate monitor. The analysis encompassed time-domain metrics (root mean square of successive differences between adjacent normal R-R intervals [RMSSD] and the standard deviation of normal-to-normal R-R intervals [SDNN]), frequency-domain metrics (high-frequency and low-frequency indices, both expressed in normalized units, and the low-frequency/ high-frequency ratio), and non-linear metrics (SD of the Poincaré plot width [SD1] and the SD of the Poincaré plot length [SD2]). HRV parameters were processed in Kubios HRV. Statistical analysis included unpaired *t*-tests, with significance set at p < 0.05.

**Results:**

The resistance-trained group demonstrated significantly higher RMSSD (75.3 [SD, 28.5] ms) and SDNN (65.8 [SD, 23.1] ms) values than the untrained group (RMSSD: 37.5 [SD, 19.6] ms; SDNN: 40.2 [SD, 14.2] ms; p < 0.01). SD1 and SD2 were also significantly higher in the resistance training group than the control group, reflecting greater parasympathetic activity.

**Conclusions:**

Long-term resistance training was associated with improved parasympathetic modulation, indicating potential cardiovascular benefits and enhanced autonomic function.

## Resumo

**Contexto:**

A variabilidade da frequência cardíaca (VFC) é um importante indicador de saúde cardiovascular. Embora os efeitos dos exercícios aeróbicos na melhora da VFC sejam bem estabelecidos, o impacto do treinamento de resistência (TR) sobre esses parâmetros ainda é pouco explorado.

**Objetivos:**

Comparar o impacto da rotina de TR nos parâmetros da VFC em jovens fisicamente ativos.

**Métodos:**

Estudo observacional com 24 participantes, sendo 12 indivíduos treinados em resistência e 12 não treinados. A VFC foi avaliada durante 15 minutos de repouso em posição supina, utilizando o monitor de frequência cardíaca Polar RS800CX^®^. A análise contemplou métricas no domínio do tempo (raiz quadrada média das diferenças sucessivas entre intervalos R-R normais adjacentes [RMSSD] e desvio-padrão dos intervalos R-R normais [SDNN]), no domínio da frequência (índices de alta frequência [HF] e baixa frequência [LF], ambos em unidades normalizadas [nu], e a razão LF/HF) e métricas não lineares (desvio-padrão da largura [SD1] e do comprimento [SD2] do gráfico de Poincaré). Os dados foram processados no *software* Kubios^®^ HRV. A análise estatística foi realizada por meio de testes *t* não pareados, com nível de significância de p < 0,05.

**Resultados:**

O grupo treinado apresentou valores significativamente maiores de RMSSD (75,3±28,5 ms) e SDNN (65,8±23,1 ms) em comparação ao grupo não treinado (RMSSD: 37,5±19,6 ms; SDNN: 40,2±14,2 ms; p < 0,01). Os valores de SD1 e o SD2 também foram significativamente superiores nos indivíduos treinados, indicando maior atividade parassimpática.

**Conclusões:**

O TR a longo prazo está associado à melhora da modulação parassimpática, sugerindo benefícios cardiovasculares e uma função autonômica aprimorada.

## INTRODUCTION

Resistance training (RT) has emerged as a critical intervention for enhancing cardiovascular health, mainly through its positive impact on heart rate variability (HRV).^1^ HRV is an important marker of cardiac autonomic modulation and the body's ability to respond to various physiological and environmental stimuli. Higher HRV parameters (reflecting parasympathetic activation) are generally associated with better cardiovascular health and a lower risk of adverse events, while lower HRV (reflecting sympathetic activation) may indicate autonomic dysfunction and increased cardiovascular risk.^2^

Recent research has investigated the relationship between RT and HRV, seeking to understand how regular exercise can influence autonomic and cardiovascular health.^3,4^ RT, characterized by exercises that increase muscle resistance through progressive loads, promotes muscle hypertrophy and beneficial cardiovascular adaptations.^5-7^ These adaptations include improved endothelial function, increased muscle mass and strength, and favorable changes in body composition, factors that contribute to overall cardiovascular health.^8^

However, most studies on HRV have focused on aerobic exercise, with less attention given to the impact of RT. This gap in the literature highlights the need for studies specifically examining the chronic effects of RT on HRV parameters.^5^ Understanding this relationship can provide valuable information for developing exercise programs that maximize cardiovascular and autonomic benefits, preventing cardiovascular diseases and improving quality of life.^9^

Therefore, the present study aimed to compare data from HRV parameters between a resistance training group and a control group. It was hypothesized that the resistance training group would have higher parasympathetic activity and lower sympathetic activity than the control group.

## MATERIALS AND METHODS

### Study design

This observational study was conducted between February and August 2024. Participants were recruited in February through flyers and social media announcements in a university campus community in Brazil. From March to April, participants came to the laboratory for anthropometric assessments and analysis of physical activity levels. Physical activity levels and habits (exercise type) were evaluated to classify participants as either resistance-trained or not. The exclusion criteria included smoking, the use of psychoactive agents (e.g., caffeine, pre-workout supplements), anabolic steroids 6 months prior to the study, and a history of cardiovascular diseases. All participants were aged 18 to 35 years and were healthy, with no comorbidities, such as diabetes mellitus, hypertension, or dyslipidemia.

The resistance-trained group consisted of individuals who had engaged in 5 to 6 weekly sessions of resistance training (RT) for at least 6 months before the experimental analysis. The control group included individuals who were physically active but had not participated in RT in the 5 years prior to the study. Physical activity levels were reported using the International Physical Activity Questionnaire-Short Form and were categorized as low, moderate, or high. Both groups reported similar weekly physical activity durations (control group: 233.8 min/week; resistance training group: 246.6 min/week).

### Participants

**Figure 1:**
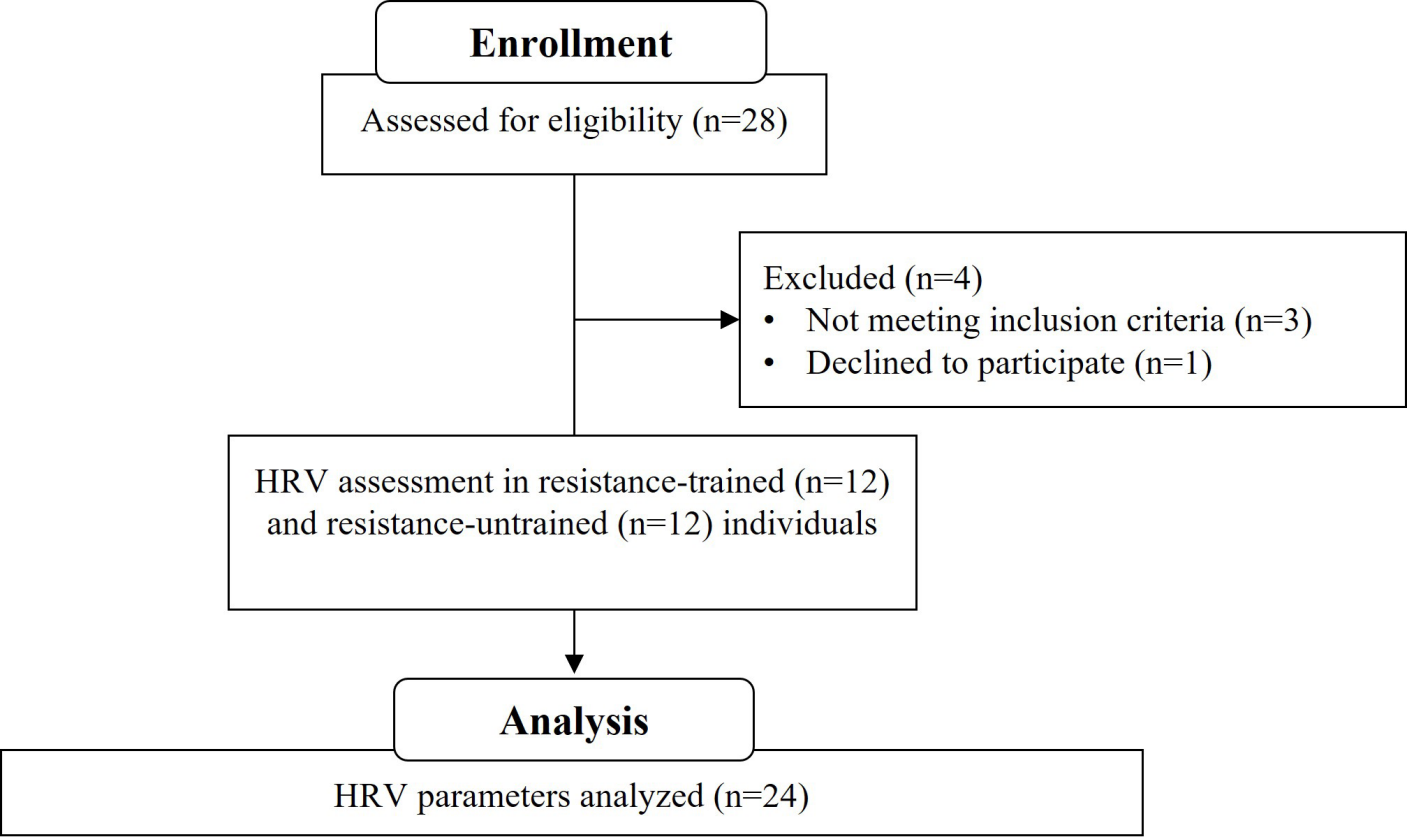
STROBE study flowchart.

Although 28 participants were initially enrolled in this study, only 24 met the inclusion criteria. These included 12 resistance-trained university students and 12 who were not (Figure 1). The baseline characteristics of the participants are presented in Table 1. Participants visited the laboratory on a single occasion to assess HRV parameters. They were instructed to abstain from physical exercise for ≥ 48 hours prior to the visit. The participants were also advised to refrain from consuming caffeine-containing foods and beverages (e.g., coffee, tea, soda, chocolate) for a minimum of 24 hours before the assessment. Female participants were evaluated during the follicular phase of their menstrual cycle to ensure consistency in the HRV analysis. All participants provided written informed consent prior to participation. The study protocol was approved by the Institutional Review Board of the Federal University of Rio de Janeiro, Campus Macaé (protocol code: 55184922.5.0000.5699; ethical approval code: 5.701.963).

**Table 1:** Demographic characteristics of the participants.

	**Resistance-trained**	**Resistance-untrained**
N (female)	12 (5)	12 (6)
Age (years)	25 ± 5	25 ± 3
Height (m)	1.71 ± 0.1	1.72 ± 0.1
Weight (kg)	73.2 ± 11.9	73.5 ± 18.1
BMI	24.9 ± 3.6	24.8 ± 4.2

BMI = body mass index. Data are expressed as mean ± SD.

### Heart rate variability measurement

To analyze HRV parameters, the participants were placed in the supine position on an examination bed in a temperature-controlled, silent room while wearing a heart rate monitor secured to the distal third of the sternum. HRV data were collected during a 15-minute rest period with a Polar RS800CX heart rate monitor, a validated device for HRV assessment. The data were subsequently transferred to a computer via the Elite HRV app 4.7.0 for analysis. A 5-minute HRV recording window was analyzed using Kubios HRV 2.0 (University of Kuopio, Finland). This software processed the HR R-R intervals, extracting relevant parameters related to HRV in time domain, frequency domain, and non-linear analysis. The root mean square of successive differences between adjacent normal R-R intervals (RMSSD) and standard deviation (SD) of the normal-to-normal R-R intervals (SDNN) were used for time domain analysis. The high-frequency (HF) index and low-frequency (LF) index, both expressed in normalized units (nu), and the low-frequency/high-frequency (LF/HF) ratio were used as frequency domain analysis. The SD of the Poincaré plot width (SD1) and the SD of the length of the Poincaré plot (SD2) were used for non-linear analysis.^9^ The RMSSD and HF are well-established as sensitive indicators of parasympathetic nervous system activity and have been used in previous studies.^7,9,10^ The SDNN represents overall autonomic modulation. The LF index is mediated by both sympathetic and parasympathetic activities. The LF/HF ratio is considered a marker of sympathovagal balance; thus, an increase in LFnu and the LF/HF ratio suggests a shift of autonomic activity to sympathetic predominance.^7,10^ The HRV parameters used in this study (RMSSD, SDNN, HF, LF, LF/HF ratio, SD1, and SD2) were based on those most examined in other studies.^6,7,9,10^

### Statistical analysis

An a priori power analysis was conducted using G*Power 3.1. The statistical power was set at 1 − *β* = 0.80, with an effect size of d = 1.2 (based on a pilot study from our laboratory). The overall significance level was set at α = 0.05. A minimum of 24 participants (12 in each group) was required to ensure sufficient power to support the null hypothesis with an acceptable level of certainty. The normality and homogeneity of data variance were examined with the Shapiro-Wilk and Levene tests, respectively. An unpaired *t*-test was performed (for independent samples) to identify significant differences in HRV parameters (RMSSD, HFnu, LFnu, SD1, SD2 index, SDNN, and LF/HF ratio) between the resistance-trained and control groups. Bootstrapping procedures (1000 re-samplings; bias accelerated and corrected 95% CI) were performed to obtain more reliable results, to correct deviations from the normality of the sample distribution, and to present a 95% confidence interval for the differences between the means.^11^ The magnitude of the effect of the RT protocol was calculated by Cohen's d, with values <0.2 considered trivial, 0.2 to <0.5 a small effect, 0.5 to <0.8 a moderate effect, and ≥ 0.8 large effect.^12^ All analyses were performed in IBM SPSS Statistics 26 (IBM, Armonk, NY, USA), and the results were expressed as means (SD).

## RESULTS

Equal variance was not assumed for the LF/HF ratio. There were no significant differences between the resistance-trained and control groups in terms of age (*t*_(22)_ = -0.41, *p* = 0.68), height (*t*_(22)_ = 0.027, *p* = 0.67), weight (*t*_(22)_ = -0.042, *p* = 0.96), and BMI (*t*_(22)_ = 0.06, *p* = 0.95).

Table 2 shows the HRV parameters of the participants. The resistance-trained group had significantly higher RMSSD (75.3 [SD, 28.5] ms) and SD1 (53.3 [SD, 20.2] ms) values than the control group, RMSSD (37.5 [SD, 19.6] ms^2^, *t*_(22)_ = 3.77, *p* = 0.001, *d* = 1.53) and SD1 (26.5 [SD, 13.8] ms, *t*_(22)_ = 3.78, *p* = 0.001, Cohen's *d* = 1.55). There was no significant difference in HFnu values between the groups (resistance-trained group: 50.3 [SD, 11.1] nu and control group: 56.5 [SD, 17.4] nu, *t*_(22)_ = -1.02, *p* = 0.33).

**Table 2:** Heart rate variability parameter data in resistance-trained and -untrained individuals.

HRV parameters		Mean ± SD	Mean difference	*Bootstrapping sample* BCa 95% confidence interval	
				Lower bound	Upper bound
RMSSD (ms)	Resistance-trained	75.3 ± 28.5**	37.75	19.78	55.82
	Control	37.5 ± 19.6			
SDNN (ms)	Resistance-trained	65.8 ± 23.1**	25.6	11.54	39.11
	Control	40.2 ± 14.2			
SD1 (ms)	Resistance-trained	53.3 ± 20.2**	26.77	14.08	39.53
	Control	26.5 ± 13.8			
SD2 (ms)	Resistance-trained	75.8 ± 27.2**	25.99	9.49	41.54
	Control	49.8 ± 16.5			
HF (nu)	Resistance-trained	50.3 ± 11.1	-6.12	-17.28	5.19
	Control	56.5 ± 17.4			
LF (nu)	Resistance-trained	47.4 ± 11.1	-10.46	-21.15	-0.17
	Control	57.8 ± 16.8			
LF/HF ratio	Resistance-trained	0.94 ± 0.4	-0.61	-1.55	0.11
	Control	1.55 ± 1.2			

** Denotes a significance difference with the control group (p<0.01). Values are presented as mean ± SD. BCa = bias accelerated and corrected; HF = high-frequency; HRV = heart rate variability; LF = low-frequency; LF/HF ratio = high-frequency/low-frequency ratio; RMSSD = root mean square of successive differences between adjacent normal R-R intervals; SD1 = SD of the Poincaré plot width; and SD2 = SD of the length of the Poincaré plot; SDNN = standard deviation of the normal-to-normal R-R intervals.

The resistance-trained group had significantly higher SDNN (65.8 [SD, 23.1] ms) and SD2 (75.8 [SD, 27.2] ms) values than the control group (SDNN 40.2 [SD, 14.2] ms, *t*_(22)_ = 3.25, *p* = 0.004, Cohen's *d* = 1.41; SD2 49.8 [SD, 16.5] ms, *t*_(22)_ = 3.25, *p* = 0.004, Cohen's *d* = 1.15). There was no significant difference in LF/HF ratio between the resistance-trained (0.94 [SD, 0.4]) and control (1.55 [SD, 1.2], *t*_(13.3)_ = -1.58, *p* = 0.18) groups. There was no significant difference in LFnu between the resistance-trained (47.4 [SD, 11.1] nu) and control groups (57.8 [SD, 16.8] nu, *t*_(22)_ = -1.79, *p* = 0.09, Cohen's *d* = 0.73) groups.

## DISCUSSION

The present study investigated how routine RT affects HRV parameters in healthy, physically active young adults. We observed that the resistance-trained group had higher RMSSD and SD1 values than controls. The resistance-trained group also had significantly higher overall autonomic modulation (SDNN). This suggests that RT can induce higher cardiac parasympathetic modulation, which is considered a cardioprotective phenomenon with relevant clinical implications.^7^

Several previous studies have demonstrated the effect of a single session of RT on HRV parameters.^13-15^ Previous studies have manipulated RT variables (rest interval length between sets, number of sets, load intensity, etc.) in an RT session to assess HRV parameters before and after RT. Although HRV parameter data after a single session of RT are relevant since they allow assessment of the post-exercise recovery status of cardiac autonomic modulation, the impact of long-term (> 4 week) RT programs is still unclear. For example, a systematic review and meta-analysis investigating the effect of RT (> 4 weeks) on HRV parameters reported that RT had minimal effects on cardiac autonomic modulation in healthy individuals.^5^ However, it is important to note that the data included in the systematic review and meta-analysis were heterogeneous, making it difficult to draw solid conclusions.^5^

Recent studies have been conducted on the chronic effect of RT programs on HRV parameters.^16,17^ Li et al.^16^ investigated the impact of an 8-week RT program on HRV parameters (SDNN and LF/HF ratio) in female college students with anxiety symptoms.^16^ After the RT intervention, there was a significant increase in SDNN and a significant decrease in LF/HF ratio values, suggesting improvement in autonomic modulation.^16^ Lin et al.^17^ evaluated the effect of 24 weeks of RT at different intensities (low-moderate intensity [50% 1 RM] and high intensity [80% 1RM]) on HRV parameters in middle-aged and older adults. Compared to baseline, the LF/HF ratio in the high-intensity group was significantly lower after the RT program.^17^ Moreover, after the RT program, the high-intensity group had significantly higher HF values than the control group.

Previous Brazilian studies have reported similar findings to the above-mentioned studies. For example, Rezende Barbosa et al.^18^ evaluated the effects of 12 weeks of RT on HRV parameters (SD1 and SD2 indices) in 29 women, finding a significant increase in SD1 and SD2 indices in the resistance-trained group compared to the control group.^18^ Their findings suggest that the RT program had a beneficial effect on autonomic modulation, characterized by increased parasympathetic activity. Similarly, Mariano et al.^19^ reported that a 10-week intervention combining 20 minutes of RT and 20 minutes of aerobic exercise three times a week led to comparable improvements in HRV parameters in both normotensive and hypertensive postmenopausal women. These findings reinforce the positive impact of RT on autonomic regulation in different populations, highlighting its potential role in cardiovascular health.

This evidence aligns with our findings, since resistance-trained individuals have a significantly higher SD1 and SD2 index, RMSSD, HFnu, and SDNN than individuals who do not engage in RT program. It is important to note that previous studies^16,17^ have essentially investigated the impact of a long-term RT program on HRV parameters compared to a non-exercise control group. In this context, it is difficult to determine whether the RT program presents a particular physiological aspect that beneficially modulates the autonomic system or whether any form of exercise can positively affect autonomic modulation. Although studies have investigated the effect of different types of exercise (RT, running, functional training) on HRV parameters, it is difficult to match exercise intensity^18,20^ for adequate comparison. The present study compared two groups of individuals: those engaged in resistance training and those not engaged. The control group (resistance untrained individuals) spent a similar amount of time in moderate-intensity cardiorespiratory exercise (e.g., jogging, running, swimming, cycling, soccer) as the resistance group spent on RT. Thus, the HRV parameters of resistance-trained individuals were not compared with sedentary individuals, as has typically been done in previous studies evaluating the impact of RT programs on HRV parameters.^16-18^

The present study provides valuable insights into cardiac autonomic response adaptations in resistance-trained individuals, who demonstrated greater parasympathetic activation at rest compared to a group of individuals who engaged in moderate-intensity cardiorespiratory exercise. A previous study has indicated that individuals with higher RMSSD and SD1 values at rest have an enhanced capacity for elevated heart rate during a maximal repetition test on the bench press machine – a resistance exercise targeting the pectoral muscles.^20^ This rapid increase in heart rate during exercise is crucial for delivering oxygen and nutrients to active muscles.^21^

### Experimental considerations

A limitation of this study was the small sample size (12 participants), which could affect the findings. However, the large effect size observed between the resistance-trained and control groups reinforces our findings, since the effect size (Cohen’s *d*) is unaffected by sample size.^12^ Another limitation is that the intensity and volume of the participants' individual RT programs were not controlled. Although such evaluation was beyond the study’s objectives, we applied International Physical Activity Questionnaire-Short Form to measure exercise frequency and intensity. Furthermore, it is noteworthy that LF/HF ratio was lower in the resistance-trained group than the control group, presenting a trend toward significance (p = 0.09) and a moderate effect size (Cohen's *d* = 0.73), which is in line with a previous study showing significantly decreased LF/HF ratio after an 8-week RT program in female college students with anxiety symptoms.^16^ The LF/HF ratio is often used as an indicator of the balance between sympathetic and parasympathetic nervous system activity; thus, when the LF/HF ratio is lower, it typically represents a shift toward greater parasympathetic activity and reduced sympathetic dominance.^16^

It is also important to note that improved cardiac autonomic regulation has significant implications for cardiovascular health, since it is linked to a lower risk of cardiovascular disease. For vascular surgeons, these findings highlight the critical importance of integrating resistance training into cardiovascular prevention and rehabilitation programs. Additionally, the observed positive effects on HRV parameters could benefit patients with vascular diseases by enhancing autonomic function and mitigating complications related to autonomic imbalance, including hypertension and heart failure.

## CONCLUSION

The present study showed that resistance-trained individuals had higher HRV parameters (RMSSD, SD1, SD2, SDNN) than controls, which suggests that long-term RT enhances parasympathetic modulation. Incorporating RT into regular exercise routines could contribute to cardiovascular disease prevention by improving cardiac autonomic regulation.
